# ICSI Does Not Improve Live Birth Rates but Yields Higher Cancellation Rates Than Conventional IVF in Unexplained Infertility

**DOI:** 10.3389/fmed.2020.614118

**Published:** 2021-02-10

**Authors:** Jianyuan Song, Tingting Liao, Kaiyou Fu, Jian Xu

**Affiliations:** ^1^The Fourth Affiliated Hospital, Zhejiang University School of Medicine, Yiwu, China; ^2^Reproductive Medicine Center, Tongji Hospital, Tongji Medical College, Huazhong University of Science and Technology, Wuhan, China; ^3^Women's Hospital School of Medicine Zhejiang University, Hangzhou, China

**Keywords:** IVF, ICSI, unexplained infertility, cancellation rates 3, live birth rate

## Abstract

**Objectives:** Unexplained infertility has been one of the indications for utilization of intracytoplasmic sperm injection (ICSI). However, whether ICSI should be preferred to IVF for patients with unexplained infertility remains an open question. This study aims to determine if ICSI improves the clinical outcomes over conventional *in vitro* fertilization (IVF) in couples with unexplained infertility.

**Methods:** This was a retrospective cohort study of 549 IVF and 241 ICSI cycles for patients with unexplained infertility at a fertility center of a university hospital from January 2016 and December 2018. The live birth rate and clinical pregnancy rate were compared between the two groups. Other outcome measures included the implantation rate, miscarriage rate, and fertilization rate.

**Results:** The live birth rate was 35.2% (172/488) in the IVF group and 33.3% (65/195) in ICSI group, *P* = 0.635. The two groups also had similar clinical pregnancy rates, implantation rates, and miscarriage rates. The fertilization rate of IVF group was significantly higher than that of ICSI group (53.8 vs. 45.7%, *P* = 0.000, respectively). Sixty-one and 46 patients did not transfer fresh embryos in IVF and ICSI cycles, respectively. Patients with IVF cycles had lower cancellation rates than those with ICSI (11.1 vs. 19.1%, *P* = 0.003, respectively).

**Conclusion:** ICSI does not improve live birth rates but yields higher cancellation rates than conventional IVF in the treatment of unexplained infertility.

## Introduction

Though the technique of directly injecting a selected spermatozoon into each oocyte was introduced for male factor infertility (1), there has been an increase in the use of intracytoplasmic sperm injection (ICSI) for all populations even without male factor. It has been reported that ICSI use has expanded from 15.4 to 66.9% during 1996–2012 in non-male factor cases (2). ICSI may give rise to an increased likelihood of fertilization, but general use of ICSI for all cases of infertility is not recommended in assisted reproductive technology (ART) (3, 4).

Unexplained infertility has been one of the indications for utilization of ICSI. Appropriate 30–40% infertile couples are diagnosed as having unexplained infertility, in whom no abnormalities are found during the fertility work-up including semen analysis, tests of ovulation, assessments of tubal patency, and the pelvic cavity (5). However, whether ICSI should be preferred to *in vitro* fertilization (IVF) for patients with unexplained infertility remains an open question. Oocyte damage is one of the potential problems with this invasive technique, which is unpredictable and unsystematic in nature (6–8). Thus, several clear end points need to be assessed if advocating the routine use of ICSI in unexplained infertility: normal fertilization rates, embryo quality, implantation rates, and live birth rates. This study aims to report clinical outcomes of patients with unexplained infertility, resulting from their first cycle of ICSI vs. conventional IVF.

## Materials and Methods

### Study Population

**Figure 1:**
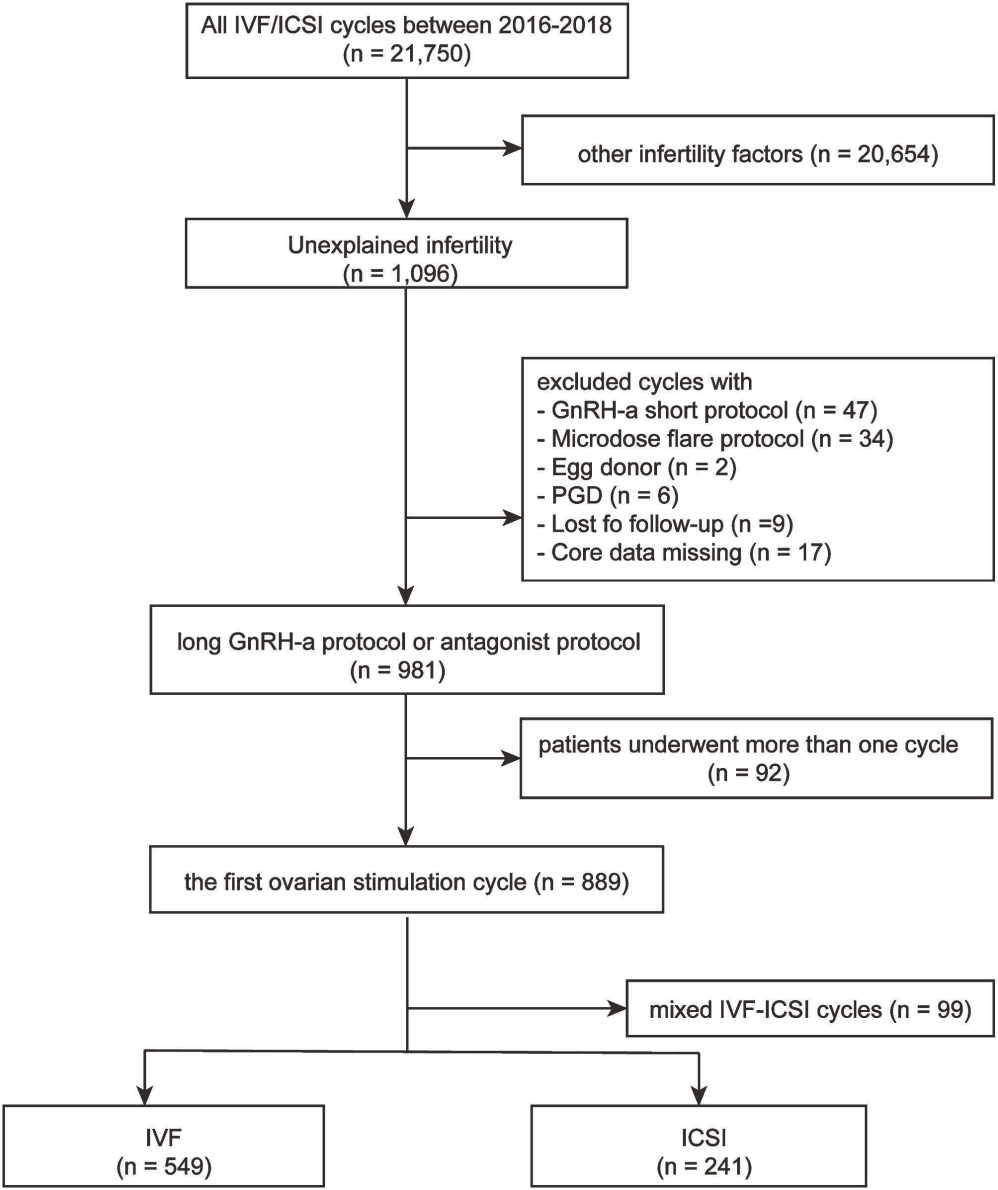
Flow chart of the study. Describing flow sheet of enrolled patients.

IVF and ICSI cycles between January 2016 and December 2018 in Women's Hospital School of Medicine Zhejiang University were screened. Clinical and laboratory information were taken from both groups and patients' data were used anonymously. The inclusion criteria included the following: (1) patients with unexplained infertility, which was defined as no abnormalities were found during the fertility work-up including semen analysis, tests of ovulation, assessments of tubal patency, and the pelvic cavity; (2) first controlled ovarian stimulation cycles; (3) IVF or ICSI; (4) long gonadotrophin releasing hormone (GnRH) agonist protocol or GnRH antagonist protocol. The exclusion criteria were: (1) rescue ICSI; (2) patients with egg donor; (3) patients with preimplantation genetic diagnosis; (4) lost to follow-up or core data missing. The flow chart of the study was shown in Figure 1. The study was approved by the Institutional Review Board at Zhejiang University, China on 22, June 2020, with the approval number (IRB-20200164-R) and was carried out in accordance with the Helsinki Declaration.

### Sample Size

Since most previous studies concluded to insignificant differences in the pregnancy rates between ICSI and conventional IVF in non-male factor or unexplained infertility patients, this study was powered to assess ICSI's effects on the fertilization outcome comparing with IVF in unexplained infertility. The sample size was calculated by statistical package in R (version 3.6.0, Austria) according to the difference in fertilization rates (53 vs. 40%) published before (9). When using a 1:2 match, to provide a two significance level of 0.05 and a power of 80%, 230 participants were required in ICSI group and 460 in IVF group.

### Treatment

Ovarian stimulation procedures: in the long GnRH agonist protocol, 0.1 mg of GnRH-a was injected daily from mid-luteal phase. Then 75–300 IU rhFSH would start 14 days later after confirming downregulation. In the GnRH antagonist protocol, administration of 200–225 IU/day rFSH (Gonal-F; MerckSerono, Geneva, Switzerland) was started on day 2 of the menstrual cycle. When the mean diameter of the dominant follicle reached was <14 mm, the GnRH antagonist Cetrotide (0.25 mg s.c., Merck-Serono, Geneva, Switzerland) was injected daily until the day of HCG administration. When two leading follicles reached a mean diameter of 18 mm, HCG 10,000 IU (HCG, Livzon Pharmaceutical Group Inc., China) was administered to trigger ovulation. Transvaginal oocyte retrieval was performed 34–36 h after HCG administration.

Standard IVF insemination and ICSI protocols: in IVF group, oocytes were inseminated by sperm with progressive motility concentrate 0.1–0.2 × 10^6^, while in ICSI group, the cumulus–oocyte complex (COC) was stripped and oocytes were inseminated by injecting a spermatozoon. In both groups, a fertilization check was performed under an inverted microscope at 16–18 h after insemination. Embryo quality was analyzed according to the Istanbul consensus workshop on embryo assessment (10). Embryos (Grade B) with 7–9 cell and less than 10% fragmentation and even symmetry were graded as good quality. Embryo transfer was performed on day 3 after fertilization.

### Definition of Clinical Outcomes

The primary outcome measures were the live birth rate and clinical pregnancy rate. The secondary outcomes included the implantation rate, miscarriage rate, and fertilization rate. The live birth rate was defined as delivery of any viable infant after 24 weeks. The clinical pregnancy was classified as the presence of an intrauterine gestational sac with fetal heart on transvaginal ultrasound 2–3 weeks after a positive pregnancy test. The implantation rate was determined by the number of gestational sacs divided by the number of embryos transferred. The miscarriage rate reflected pregnancy loss before the 24th gestational week. The fertilization rate was defined as the percentage of two visualized pronuclei (2PN) per the total number of retrieved oocytes.

### Statistical Analysis

Categorical data were summarized as frequencies and percentages and were analyzed using a Chi-square test. Continuous data were presented as mean ± standard deviation (SD) and were assessed using analysis of variance (ANOVA). The differences in clinical outcomes per cycle between the two groups were compared using Pearson's chi-squared or Fisher exact test. *P* < 0.05 was considered statistically significant. All statistical analyses were performed with the Statistical Package for Social science version 23.0 (SPSS, Inc.).

## Results

### Baseline Characteristics

Five hundred and forty-nine patients who underwent IVF and 241 underwent ICSI between January 2016 and December 2018 were included. Characteristics of included patients were summarized in Table 1. The average age of the 549 women enrolled in the IVF group was 31.2 ± 3.7 years (mean ± SD), and 241 women in the other group was 31.2 ± 4.0 years, *P* = 0.951. No statistically significant difference was found in maternal age, years of infertility, follicle stimulating hormone (FSH), antral follicle count (AFC), body mass index (BMI), or type of infertility between the two groups.

**Table 1:** Baseline characteristics of the patients.

	**IVF**	**ICSI**	***P***
Number of patients	549	241	
Age (years)	31.2 ± 3.7	31.2 ± 4.0	0.951
infertility (years)	3.7 ± 2.3	3.8 ± 2.5	0.492
FSH (mIU/ml)	6.5 ± 1.6	6.4 ± 1.5	0.290
AFC	12.4 ± 6.0	13.0 ± 6.2	0.244
BMI	21.57 ± 2.6	21.3 ± 2.5	0.212
Primary infertility	330 (60.1%)	160 (66.3%)	0.094

*Data are expressed as mean ± SD or number (percentage)*.

*IVF, in vitro fertilization and embryo transfer; ICSI, intracytoplasmic sperm injection; FSH, follicle stimulating hormone; AFC, antral follicle count; BMI, body mass index*.

### Controlled Ovarian Stimulation Procedures and Clinical Outcomes

As shown in Table 2, the number of 2PN was significantly higher in IVF group than that in ICSI group. Other results including serum hormone levels on the HCG day, the number of oocytes retrieved, the number of embryos transferred, and variable blastocyst frozen were comparable between the two groups.

**Table 2:** Controlled ovarian stimulation and clinical outcomes.

	**IVF**	**ICSI**	***P***
**Stimulation characteristics**			
Number of Patients	549	241	
Ovarian stimulation protocol			0.320
GnRH agonist protocol	384	160	
GnRH antagonist protocol	165	81	
Estradiol (pg/ml)	2,298.4	2,671.4	0.062
Progesterone (ng/ml)	0.9 ± 0.3	0.9 ± 0.6	0.666
LH (mIU/ml)	1.0 ± 0.7	1.1 ± 0.6	0.262
Number of oocytes retrieved	12.2 ± 6.1	12.7 ± 6.3	0.271
Endometrial thickness (mm)	11.6 ± 2.4	12.0 ± 2.8	0.092
Number of 2PN	6.5 ± 4.4	5.8 ± 4.4	0.028
Number of embryo transfer			0.672
1	171 (35.1%)	65 (33.3%)	
2	317 (65.0%)	130 (66.7%)	
Number of blastocyst frozen	1.6 ± 0.8	2.1 ± 1.2	0.058
**Clinical outcomes**			
Cancellation rate (%)	11.1 (61/549)	19.1 (46/241)	0.003
Fertilization rate (%)	53.8	45.7	0.000
Live birth rate (%)	35.2 (172/488)	33.3 (65/195)	0.635
Clinical pregnancy rate (%)	45.9 (224/488)	47.2 (92/195)	0.742
Implantation rate (%)	35.0 (282/805)	33.8 (110/325)	0.705
Miscarriage rate (%)	23.2 (52/224)	29.3 (27/92)	0.253

*Data are expressed as mean ± SD or number (percentage)*.

*LH, luteinizing hormone; PN, two visualized pronuclei; IVF, in vitro fertilization and embryo transfer; ICSI, intracytoplasmic sperm injection*.

The live birth rate was 35.2 (172/488) in the IVF group and 33.3 (65/195) in ICSI group, *P* = 0.635 (Table 2). The two groups also had similar clinical pregnancy rates, implantation rates, and miscarriage rates.

The fertilization rate of IVF group was significantly higher than that of ICSI (53.8 vs. 45.7%, *P* = 0.000, respectively). Patients with IVF had lower cancellation rate than those with ICSI [11.1% (61/549) vs. 19.1% (46/241), *P* = 0.003].

### Reasons for Cancellation of Fresh Embryo Transfer

**Figure 2:**
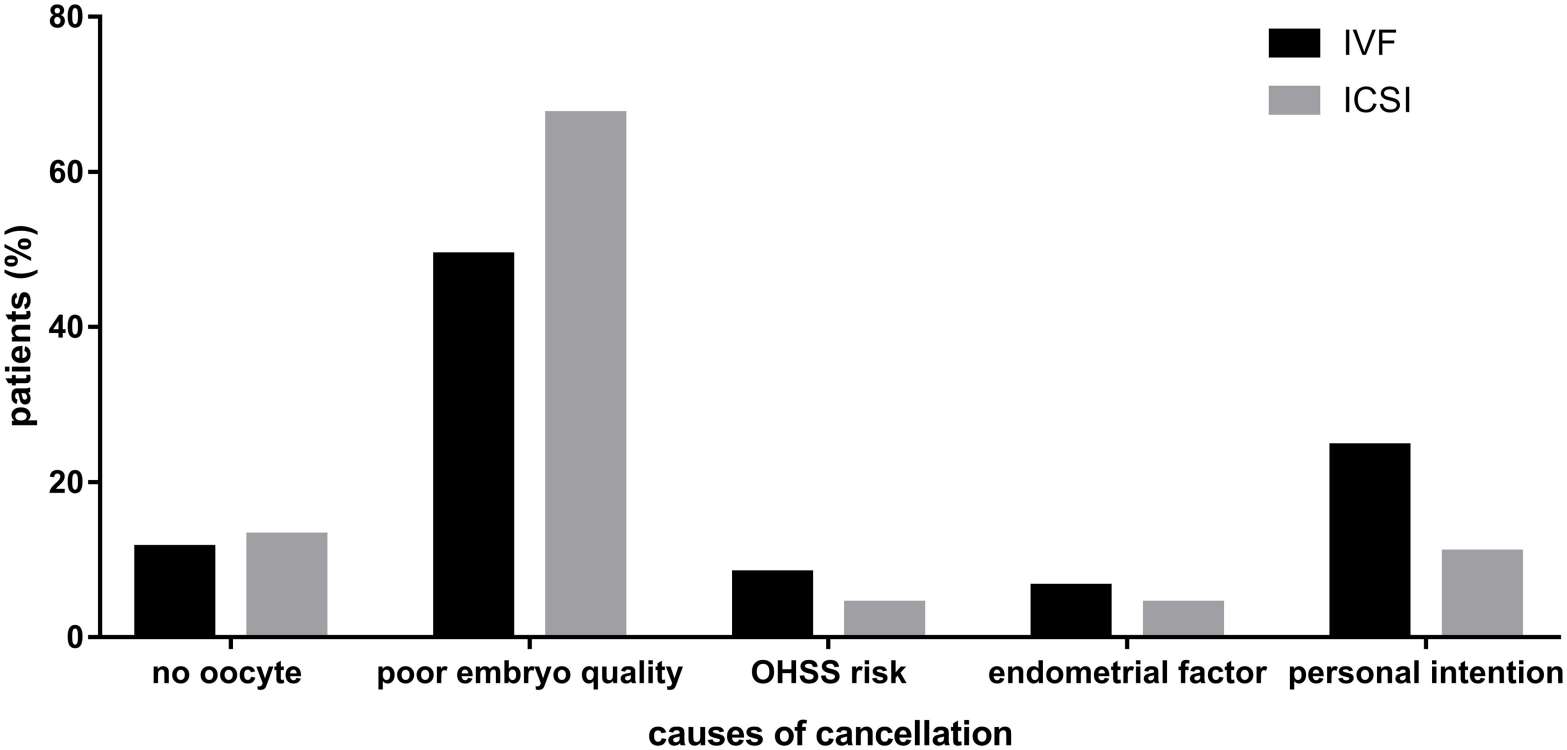
Causes of cancellation in IVF and ICSI cycles. Describing the percentage of different causes of cancellation in two groups.

In total, 61 and 46 patients did not transfer fresh embryos in IVF and ICSI cycles, respectively. Causes of cancellation in IVF cycles were no oocyte retrieved (*n* = 7), poor embryo quality (*n* = 30), ovarian hyper-stimulation syndrome (OHSS) risk (*n* = 5), endometrial factor (*n* = 4), and personal intention (*n* = 15). The number of these causes in ICSI cycles was 6, 31, 2, 2, and 5, respectively. The percentage of different causes of cancellation in two groups was shown in Figure 2.

## Discussion

The retrospective cohort study provided the first-time evidence that ICSI did not improve the live birth rate in comparison to IVF in couples with unexplained infertility. In addition, our results demonstrated the higher cancellation rates in ICSI cycles than that of IVF and the relative causes.

During ICSI procedure, a single spermatozoon is injected into the oocyte cytoplasm, which was first introduced to cases with severe male factor infertility. The indication for ICSI has embraced a larger group of infertile couples including those with unexplained infertility for several decades. Up to 30–40% of couples coming for fertility treatment will ultimately diagnosed with unexplained infertility (11). A recent study predicting the chances of having a baby in patients with unexplained infertility proved that active management was associated with higher rates of live birth than expectant management (12). Active clinical treatments for explained infertility included ovarian stimulation, intrauterine insemination (IUI), IVF, and ICSI. But evidence of differences in live birth among these interventions was insufficient (13).

Conclusions on the benefits of ICSI in patients with unexplained subfertility were unclear because there were no data from randomized clinical trials comparing live birth rates and adverse events (14). In addition, ICSI is more invasive, more costly and more time-consuming than IVF. The aim of our study was to investigate whether ICSI yielded better clinical outcomes compared with IVF in couples with unexplained infertility. Interesting, the rates of live birth, clinical pregnancy, and miscarriage were achieved similar by two different methods of fertilization in ART. This finding is consistent with previous studies. A prospective randomized trial of conventional IVF vs. ICSI in 60 patients with unexplained infertility suggested that there were no significant differences in any of the outcome measures between them–fertilization rate, implantation rate, embryo quality, or clinical pregnancy rate (15). Another study which applied ICSI and routine IVF randomly on sibling oocytes during the first cycle in seventy couples with unexplained infertility concluded that ICSI was not superior to IVF as an insemination technique in most cases (16). The number of patients enrolled in the former study was 60 and in the latter study was 70 and no information was available regarding cancellation rates. Several more recent researches reported the similar conclusions that ICSI did not confer any benefit in improving the clinical pregnancy or live birth outcome of the embryo transferred cycles when compared to IVF (17–19), but all kinds of infertility diagnosis except for male factor infertility were included in these studies.

A meta-analysis displayed that patients with well-defined unexplained infertility might benefit from use of ICSI to fertilize all oocytes (20). The difference between this and our findings is probably attributable to the different populations. We calculated the fertilization rates of all included ovarian stimulation cycles and total number of oocytes, while most previous studies took into account only the fresh embryo transfer cycles or oocytes inseminated. In other words, they did not consider those population who did not obtain good-quality embryos, which was also the potential explanation for the higher cancellation rates in the ICSI group than IVF. It's noteworthy that ICSI resulted in higher cancellation rates of fresh embryo transfer than IVF, providing more financial and emotional burden on patients for the same pregnancy results. One of the main reasons was the higher proportion of patients without good-quality embryos to transfer in ICSI cycles, which were shown in Figure 2. The hypothesis is supported by some previous studies. For example, Luna et al. (21) mentioned in the discussion part of their study that different results were encountered when analyzing fertilization rates of all oocytes or only mature oocytes. Similarly, a study of 91 patients revealed that average percentage of oocytes fertilized in ICSI was lower than that in conventional IVF (40 vs. 53%, *P* = 0.000, respectively) when calculating total oocytes retrieved, resulting that ICSI yielded lower percentage of available embryos than IVF [76.7 vs. 84.8%, respectively (9)].

Patients in the IVF group had more 2PN and higher fertilization rates than those in ICSI group although the two groups had similar number of oocytes retrieved (Table 2). Possible reasons for this difference may be the theoretical concerns of increased chromosomal anomalies, molecular disturbances, DNA methylation changes, imprinting disorders, low implantation potential, and oocyte damage brought by invasiveness of the technique (22–29). The inclusive criteria of our study population is unexplained infertility. The underlying cause of infertility in these couples is unknown, which suggests that, with the advancement of diagnostic technology and the discovery of more diagnosis, the effect of different treatments on these patients may change.

The main limit of our study was the retrospective design from a single medical center. These conclusions warrant further confirmation by other larger, prospective researches.

## Conclusion

ICSI does not improve live birth rates but yields higher cancellation rates than conventional IVF in the treatment of unexplained infertility.

## Data Availability Statement

The original contributions presented in the study are included in the article/supplementary material, further inquiries can be directed to the corresponding author/s.

## Ethics Statement

The study was approved by the Institutional Review Board at Zhejiang University, China and was carried out in accordance with the Declaration of Helsinki.

## Author Contributions

The study was designed by JX. Material preparation, data collection, and analysis were performed by JS, TL, and KF. The first draft of the manuscript was written by JS. All authors read and approved the final manuscript.

## Conflict of Interest

The authors declare that the research was conducted in the absence of any commercial or financial relationships that could be construed as a potential conflict of interest.
